# Supplementary material for: Asymptomatic osteolysis as a risk factor for cardiovascular disease after total hip arthroplasty: A retrospective cohort study
Source: PLoS One. 2025 Dec 30;20(12):e0338676. doi: 10.1371/journal.pone.0338676 (PMC12752981; doi:10.1371/journal.pone.0338676)
Supplement: Attachment — Submitted filename: Rebuttal_letter_Agata_Sara (2).docx [file pone.0338676.s002.docx]

May 17, 2025

Agata Rysinska

Manuscript PONE-D-24-55835 Response to Reviewer

Dear Nan Jiang,

Thank you for giving us the opportunity to submit a revised draft of the “Asymptomatic Osteolysis as a Risk Factor for Cardiovascular Disease After Total Hip Arthroplasty:

A retrospective cohort study” manuscript for publication in the PLOS ONE. We appreciate the time and effort that you and the reviewers dedicated to providing feedback on our manuscript and are grateful for the insightful comments on and valuable improvements to our paper. Please see below our comments and answers.

1:

All raw data is uploaded as Supporting Information files

2:

Regarding variability in surgical procedures since the surgeries were performed by different surgeons at various times and under different conditions-if potential individual surgical errors or difference I technique could have influenced the outcomes.

*Answer: Thank you for this relevant question.*

*We have not the surgery reports from the cases.*

*A total hip arthroplasty is for an orthopedic surgeon a routine surgery, and a surgeon under training is not leaved to perform surgery alone until he or she is assessed by a more experienced surgeon to be ready to do so. It might though influence the results.*

3:

Considering the limited number of events, is the Cox model adequate for this dataset?

*Answer: You have raised an important question*

*Due to the nature of the dataset we believe that another model would not change the conclusion of our study.*

4:

Patients who died before the study inclusion were not accounted for possibly underestimating the actual cardiovascular risk. ECG was used for cardiovascular assessment-it is not the most reliable predictor of future cardiovascular events. More advanced cardiac imaging or functional tests could have provided stronger evidence. The study measured hs-CRP, leukocytes, and lipid levels once, missing potential variations over time. The study does not fully control confounders for lifestyle factors, comorbidities, or other medications that may affect cardiovascular outcomes.

Answer: We agree with your assessment.

*We have not adjusted for patients that died before the study-this could underestimate the risk of death due to cardiovascular causes. ECG might today not be the best reliable predictor for future cardiovascular events, but is readily available and entails no risks. Methods like stress echocardiography, myocardial scintigraphy or computed tomography of the coronary arteries might be used in future studies. The blood tests were collected once-missing potential variations-which is an inherent limitation of a cross-sectional study. Lifestyle factors or comorbidities that may affect cardiovascular outcomes are not always mentioned in medical records which could affect the results. We have, however, included information on the prespecified variables whenever available.*

Sincerely

Agata Rysinska
