# Supplementary material for: Asymptomatic osteolysis as a risk factor for cardiovascular disease after total hip arthroplasty: A retrospective cohort study
Source: PLoS One. 2025 Dec 30;20(12):e0338676. doi: 10.1371/journal.pone.0338676 (PMC12752981; doi:10.1371/journal.pone.0338676)
Supplement: Attachment — Submitted filename: Rebuttal_letter_Oct2025.docx [file pone.0338676.s003.docx]

Oct 6^th^, 2025

Agata Rysinska

PONE-D-24-55835R1

Asymptomatic Osteolysis as a Risk Factor for Cardiovascular Disease After Total Hip Arthroplasty: A retrospective cohort study

Response to Reviewer

Dear Nan Jiang,

Thank you for giving us the opportunity to submit a revised draft of the “Asymptomatic Osteolysis as a Risk Factor for Cardiovascular Disease After Total Hip Arthroplasty:

A retrospective cohort study” manuscript for publication in the PLOS ONE. We appreciate the time and effort that you and the reviewers dedicated to providing feedback on our manuscript and are grateful for the insightful comments on and valuable improvements to our paper. Please see below our comments and answers.

Reviewer #3: I am honored to have the opportunity to review this first study investigating the incidence of CVD in patients with or without asymptomatic osteolysis.

**1.** Inclusion Criteria for Study Population The manuscript states that patients treated with uncemented THA with metal-on-polyethylene articulation were considered eligible for inclusion. Were ceramic-on-polyethylene and other bearing combinations excluded? If so, what was the rationale for this exclusion? Similarly, what was the reasoning behind excluding cemented cups and cemented stems?

**Response:** Thank you for this interesting question.

Ceramic heads tend to produce less polyethylene wear than metal heads-less biological provocation to activate macrophages, leading to bone resorption, osteolysis and possibly aseptic loosening. Cemented stems and cups were excluded since cementless components historically had higher rates of cup revision for aseptic loosening. Also uncemented cups had a larger rate of visible periacetabular osteolysis.

**2.** Exclusion of Bisphosphonate Users Could you please clarify the rationale for excluding patients who were using bisphosphonates?

**Response:** Bisphosphonates suppress osteoclast mediated bone resorption and could therefore be a potent confounder for development of periprosthetic osteolysis.

**3.** Baseline Characteristics and Statistical Adjustment The results indicate that "the proportion of patients with previous myocardial infarction was higher in the osteolysis group." Was this baseline imbalance ultimately adjusted for in the final analysis? Is the adjusted hazard ratio of 1.6 (95% CI, 0.5-5.8) the value after adjusting for this and other confounders? I apologize for my limited comprehension, but I would appreciate clarification on this point.

**Response:** Yes. The adjusted hazard ratio of 1.6 (95% CI 0.5–5.8) is the result after adjusting for age, sex, and pre-existing cardiovascular disease (including prior myocardial infarction).

**4.** Sample Size and Study Conclusions The study found no significant differences between groups in inflammatory markers (hs-CRP, leukocyte count), lipid levels, or frequency of ECG abnormalities, and the authors attributed this to insufficient sample size. If this is indeed the case, should publication be deferred until a larger sample size can be obtained? I believe there is a substantial possibility that the true conclusion might be that asymptomatic osteolysis is not associated with CVD development. This alternative interpretation should be acknowledged and discussed. Furthermore, I suggest that the value of this paper would be enhanced by addressing how large a sample size would be needed to definitively conclude "no association" and providing your expert opinion on this matter. Such discussion would provide important guidance for future research design and help readers better interpret the clinical significance of these findings.

**Response:** We performed a post-hoc feasibility calculation for a time-to-event analysis using Schoenfeld’s method. Assuming a two-sided α=0.05, power=80%, exposure prevalence of 24% (osteolysis), and a HR of 1.6, the required number of outcome events would be approximately 195. Translating events to total sample size depends on the anticipated cumulative event rate over follow-up: with event rates of 12%, 15%, and 20%, the corresponding total sample sizes would be ~1,620, ~1,300, and ~974 participants, respectively. We have added this fact to the discussion.

Reviewer #4: I appreciate the effort made by the authors in this interesting study As an observational retrospective study there are some flaws

**1:**The definite association between periprosthetic osteolysis and the resulted inflammatory response that causes CVS and cardiac disease has not been proven.

**Response:** Thank You for this true remark.

This was the material that was possible to do research on from Swedish Hip Arthroplasty Register. To prove the link, it would be needed to show temporality, biological plausibility, replication and that inflammation mediates the effect. Research would need to combine different types of research. For example:

A: Mechanistic lab work (in vitro/ animal models) to show that wear particles cause inflammation that causes vascular damage.

B: Large human observational studies (prospective cohorts) to show association and temporality.

C: Interventional evidence (anti-inflammatory or antiparticle strategies) to show reduction in both inflammation/osteolysis and cardiovascular outcomes.

D: Causal interference methods to strengthen casual claims.

**2:** The multifactorial etiology of cardiovascular disease makes an obvious difficulty of establishing correlation between osteolysis and CVD.

**Response:**

For several reasons, for example methodological, biological and epidemiological. Both conditions have shared risk factors and therefore confounding. This makes it difficult to establish correlation between periprosthetic osteolysis and cardiovascular disease. Both are common in older populations. Age, obesity, sedentary lifestyle-all increased risk of both. This makes it difficult to explain if osteolysis itself is associated with CVD or if both conditions share similar risk profile. Even with statistical adjustments residual confounding still often remains.

Another factor is that there are different primary systems and mechanisms. Periprosthetic osteolysis is a localized bone pathology around prosthetic implants driven by mainly inflammatory responses to wear particles (debris from polyethylene, metal or ceramic).

CVD on the other hand is a systemic vascular disease influenced by endothelial dysfunction due to inter alia hypertension, inflammation and endothelial dysfunction.

**3:** It is very difficult to find matched patients’ characteristics in each group with many variables that will affect the result.

**Response:**

Temporality and timing: periprosthetic osteolysis often develops many years after implantation, and it is a subclinical condition without pain, while CVD can predate or develop in parallel.

Establishing weather periprosthetic osteolysis precedes or contributes to CVD requires longitudinal follow-up studies with repeated imaging and outcome capture which is expensive and logistically difficult. Imaging requires CT analysis, not a routine method.

CVD is defined in different ways (clinical events, imaging, biomarkers), misclassification of either exposure or outcome weakens correlation.

**4:** The small number of patients studied is another weak point.

**Response:** Revision surgery for osteolysis is relatively uncommon compared with primary total hip or knee surgeries due to osteoarthritis. Large cohorts and long follow up time are needed to capture both exposures and outcomes which makes studies costly and underpowered.

**5:** The mere comparison made for the same patients before surgery and at least 10 years after surgery is invalid as the risk of CVD increases with age.

**Response:** Thank You for a very intriguing question.

Aging is an independent risk factor. With increasing age, the risk of CVD rises regardless of whether they had THA surgery. Time itself is a confounder, during the 10+years other factors as lifestyle changes, new comorbidities and/or medications could affect the CVD risk

Comparing “before THA” when patients are younger 10+years later doesn´t isolate the effect of THA, it just shows that the risk for CVD increases with higher age.

Without controlling for aging and confounding by time it is hard to explain weather the risk for CVD is because of the THA or due to natural ageing and disease progression.

Sincerely

Agata Rysinska
