# Supplementary material for: Asymptomatic osteolysis as a risk factor for cardiovascular disease after total hip arthroplasty: A retrospective cohort study
Source: PLoS One. 2025 Dec 30;20(12):e0338676. doi: 10.1371/journal.pone.0338676 (PMC12752981; doi:10.1371/journal.pone.0338676)
Supplement: Attachment — Submitted filename: Rebuttal_letter_NOV10.docx [file pone.0338676.s004.docx]

Nov 10^th^, 2025

Agata Rysinska

PONE-D-24-55835R1

Asymptomatic Osteolysis as a Risk Factor for Cardiovascular Disease After Total Hip Arthroplasty: A retrospective cohort study

Response to Reviewer

Dear Nan Jiang,

Thank you for giving us the opportunity to submit a revised draft of the “Asymptomatic Osteolysis as a Risk Factor for Cardiovascular Disease After Total Hip Arthroplasty:

A retrospective cohort study” manuscript for publication in the PLOS ONE. We appreciate the time and effort that you and the reviewers dedicated to providing feedback on our manuscript and are grateful for the insightful comments on and valuable improvements to our paper. Please see below our comments and answers.

6. Review Comments to the Author

Reviewer #3: It is a great honor to have the opportunity to review this valuable manuscript.

The authors state that they "previously showed an increased long-term risk of cardiovascular-related mortality in patients treated with THA [16], and an increased relative risk of cerebrovascular events (CVE) in patients with osteoarthritis who received THA and subsequently underwent revision surgery due to loosening of the prosthesis [17]. Similarly, cardiovascular mortality following total knee arthroplasty exceeds that of the general population after 10 years [18]."

I was completely unaware of these findings and found them highly informative. However, I am disappointed that the direct relationship could not be statistically demonstrated in this study. I would encourage the authors to consider whether statistical significance could be achieved through increased sample size or more rigorous matching.

**Action:**

An increased sample size would improve statistical analysis, ton increase the current study is ouside of the scope of this study. We do however believe that our results could be useful in the building of future hypotheses and meta-analyses. A more rigorous matching on variables that plausibly can affect both conditions.

Adults over 50 years undergoing total hip or knee arthroplasty surgery with baseline imaging. Exclusion criteria would be pre-existing advanced CVD, known active infection and immunosuppression that will confound inflammatory markers.

Baseline cardiovascular risk such as history of CVD, hypertension, diabetes, hyperlipidemia, smoking and BMI.

Medications, baseline statins, antihypertensives, antiplatelets and anticoagulants.

Multicenter study can provide implant heterogeneity. Implant type, material, fixation methods (exact match or cluster).

The authors also state that "the absence of notable differences in this study does not conclusively negate the possibility of osteolysis serving as a causal factor for CVD, as the chronic low-grade inflammation in osteolysis could hypothetically contribute to development of atherosclerosis." In this regard, I wonder whether it would be possible to examine the association between atherosclerotic burden and the presence or absence of osteolysis.

**Action:**

To establish association between atherosclerosis and periprosthetic osteolysis is difficult for many reasons: scientific, methodological and biological.

The primary systems and mechanisms differ.

*Atherosclerosis* is a systemic vascular disease influenced by lipid metabolism, endothelial dysfunction, hypertension and inflammation.

*Periprosthetic osteolysis* is bone pathology locally around prosthetic implants driven by inflammatory responses to wear particles (polyethylene, metal or ceramic debris).

Both involve inflammation, both are distinct and overlap only partially.

There are similar confounding mechanisms, due to similar age-related risk factors such a age, obesity, diabetes, smoking, inactivity etc. The shared risk factors make it difficult to determine if there is a direct relationship between atherosclerosis and periprosthetic osteolysis or if both conditions occur more often in older patients with comorbidities.

Another challenge is measurement and detection. Both conditions can occur silently for many years before being detected, therefore this mismatch in time and diagnostic variability make longitudinal correlation difficult. Standardized biomarkers or imaging endpoints are lacking for both processes currently. Cardiovascular studies don´t collect data on implant-related bone changes, and similarly: studies on periprosthetic osteolysis focus on orthopedic measurable outcomes-not systemic health. The heterogeneity in study designs limits the ability to establish association.

Prospective human studies to test this association would require large, long-term cohorts, costly and difficult.

The manuscript would benefit from including representative CT images: at least one demonstrating periacetabular osteolysis and one without osteolysis.

**Action:**

Due to the restrictions of the ethical permit, we are unable to provide the requested CT images.

Finally, regardless of whether an association between osteolysis and CVD is confirmed, the manuscript would provide greater clinical value if the authors could offer recommendations for orthopedic surgeons regarding strategies to reduce CVD risk in THA patients during long-term follow-up.

**Action:**

Should this association be proven, surgeons should take care to include and optimize specific follow up surveillance protocols regarding CVD in these patient populations. However, these protocols should be the subject of interdisciplinary collaboration between orthopaedic surgeons, cardiologists and radiologists. We have adjusted the manuscript accordingly.

Sincerely

Agata Rysinska
